# Psychosocial risk factors among nurses at Sahloul University Hospital in Sousse (Tunisia)

**DOI:** 10.1192/j.eurpsy.2025.1461

**Published:** 2025-08-26

**Authors:** J. Ben Khelifa, N. Belhadj Chabbah, F. Chelly, I. Fki, C. Sridi, N. Ben Ghadha, S. Ksibi

**Affiliations:** 1 1- Department of Occupational Medicine, Sahloul University Hospital; 2 2- University of Sousse, Faculty of Medicine Ibn Al Jazzar; 3 3- Research laboratory LR 19SP03, Sousse, Tunisia

## Abstract

**Introduction:**

Nursing staff occupy a profession that requires significant mental, emotional, and affective demands. These caregivers are particularly vulnerable to psychosocial risks that can have significant impacts on their mental and physical health, as well as on the quality of care they provide.

**Objectives:**

To describe the psychosocial risk factors at work among nurses at SAHLOUL university hospital.

**Methods:**

This is a descriptive cross-sectional study conducted among the nursing staff of SAHLOUL university hospital. Data was collected using a self-administered questionnaire. Data analysis was performed using SPSS 26 program.

**Results:**

A total of 95 nurses participated in the study. Almost all the nurses surveyed (95.8%) reported that their work constitutes a significant mental load. More than one-third of respondents (34.7%) frequently faced cases of death during their work. Most of the nurses surveyed (93.7%) felt demotivated regarding their work. Just over half of the nurses surveyed (51.6%) reported having been victims of a violent situation from a patient during their professional career. Regarding the results relative to the Karasek scale, we note that our population tends to utilize skills more than to be autonomous (35.56 vs. 34.36). Social support is generally low among our study population with an average score of 25.37±2.57.

**Conclusions:**

There is psychosocial, and particularly professional, repercussions on the psychological state of healthcare personnel, which means that care workers’ mental health needs to be addressed. Occupational health services must detect the suffering of care workers and improve the psychosocial environment.

**Disclosure of Interest:**

None Declared

